# Hormone Disruption by PBDEs in Adult Male Sport Fish Consumers

**DOI:** 10.1289/ehp.11707

**Published:** 2008-07-24

**Authors:** Mary E. Turyk, Victoria W. Persky, Pamela Imm, Lynda Knobeloch, Robert Chatterton, Henry A. Anderson

**Affiliations:** 1 Division of Epidemiology and Biostatistics, School of Public Health, University of Illinois at Chicago, Chicago, Illinois, USA; 2 Wisconsin Division of Public Health, Bureau of Environmental Health, Madison, Wisconsin, USA; 3 Departments of Obstetrics and Gynecology and Physiology, Feinberg School of Medicine, Northwestern University, Chicago, Illinois, USA

**Keywords:** brominated flame retardants, hormone, PBDEs, sex hormone binding globulin, sport fish, testosterone, thyroglobulin antibodies, thyroid hormone

## Abstract

**Background:**

Persistent pollutants, such as polychlorinated biphenyls (PCBs), affect endocrine function. Human exposure to polybrominated diphenyl ethers (PBDEs), which are similar in structure to PCBs, has increased recently, but health effects have not been well studied.

**Objectives:**

Our goal in this study was to determine whether PBDE body burdens are related to thyroid and steroid hormone levels, thyroid antibodies, and thyroid disease in a cohort of frequent and infrequent adult male sport fish consumers.

**Methods:**

We tested serum from 405 adult males for PBDE congeners, PCB congeners, testosterone, sex-hormone–binding globulin (SHBG), SHBG-bound testosterone, thyroglobulin antibodies, and the thyroid hormones thyroxine (T_4_), triiodothyronine (T_3_), thyroid-stimulating hormone (TSH), and T_4_-binding globulin (TBG). We collected data on demographics, fish consumption, medical diseases, and medication use.

**Results:**

The median sum of PBDEs was 38 ng/g lipid. In 308 men without thyroid disease or diabetes, PBDEs were positively related to measures of T_4_ and reverse T_3_ and inversely related to total T_3_ and TSH. PBDEs were positively related to the percentage of T_4_ bound to albumin, and inversely related to the percentage of T_4_ bound to TBG. Associations of BDE congeners with hormones varied. BDE-47 was positively associated with testosterone levels. Participants with PBDEs over the 95th percentile were more likely to have thyroglobulin antibodies, although high PBDE exposure was not associated with thyroid disease. PBDE effects were independent of PCB exposure and sport fish consumption.

**Conclusions:**

PBDE exposure, at levels comparable with those of the general U.S. population, was associated with increased thyroglobulin antibodies and increased T_4_ in adult males.

Polybrominated diphenyl ethers (PBDEs) are used as flame retardants in electronic equipment, home furnishings, textiles, and construction materials. They are similar to polychlorinated biphenyls (PCBs) in structure and in their persistence and bioaccumulative properties (Birnbaum and Staskal 2004). Over the last 20 years, PBDE levels have increased in human samples, whereas PCBs have declined (Schecter et al. 2005).

Because PBDEs are similar in structure to thyroxine (T_4_) and triiodothyronine (T_3_) (Hamers et al. 2006), concerns have been raised regarding their effect on thyroid function, which is regulated by the hypothalamo–pituitary–thyroid axis and influences development and gene expression in vertebrates (Zoeller et al. 2007). Reduction of circulating thyroid hormone is compensated for by release of thyroid-releasing hormone from the hypothalamus, which in turn increases thyroid-stimulating hormone (TSH) release from the pituitary, ultimately stimulating thyroid hormone production. T_4_ and T_3_ are transported to peripheral tissues bound to proteins, primarily T_4_-binding globulin (TBG), but also to albumin and transthyretin (TTR). TBG production is stimulated by estrogen and inhibited by testosterone. T_4_ is the major hormone produced by the thyroid. Some T_3_ is produced directly by the thyroid, but most is derived from peripheral deiodination of T_4_. T_3_ and T_4_ are primarily metabolized by deiodination to diiodothyronine and reverse T_3_ (rT_3_), with some metabolism through glucuronidation, sulfonation, and other pathways. This complex system is vulnerable to disruption by a variety of chemicals through changes in hormone production, transport, and/or metabolism (Zoeller et al. 2007).

Biologic effects of PBDEs in rodents are similar to those of PCBs, with increased risks for reproductive and endocrine disruption (Ellis-Hutchings et al. 2006; Lilienthal et al. 2006; Stoker et al. 2004; Zhou et al. 2002), and neurodevelopmental problems (Kuriyama et al. 2005). In humans, PCBs have been associated with disruption of thyroid hormone homeostasis (Langer et al. 2007; Persky et al. 2001; Turyk et al. 2007), but the effects of PBDEs on thyroid hormones have been investigated only in a few smaller studies (Bloom et al. 2008; Hagmar et al. 2001; Julander et al. 2005; Yuan et al. 2008).

In 2001, we reported that PCBs were associated with lower levels of T_4_ and free T_4_ index in women and T_4_ and sex-hormone–binding globulin (SHBG)-bound testosterone in men from a cohort of frequent and infrequent Great Lakes fish consumers (Persky et al. 2001). In 2003, we invited participants from the original cohort to participate in a follow-up study to explore potential mechanisms by which PBDEs, PCBs, and *p,p*′-diphenyl-dichloroethene (DDE) might be affecting thyroid hormone balance. In addition to the standard hormones (free and total T_4_ and T_3_, as well as TSH), we explored via additional laboratory parameters specific mechanisms of action suggested by laboratory studies, such as changes in transport by serum-binding proteins (Hallgren et al. 2001; Hamers et al. 2006) and increase in thyroglobulin antibodies (Langer et al. 2007). In this study we explored the relationship of PBDE exposure with hormone homeostasis, thyroglobulin antibodies, and thyroid disease in men. Associations of thyroid hormones with PCB congeners and DDE will be reported separately.

## Materials and Methods

### Study participants

We invited a cohort of 4,206 frequent and infrequent consumers of Great Lakes fish established during the early 1990s (Hanrahan et al. 1999) to participate in a follow-up study. Information on fish consumption, medical diseases, and use of prescription and over-the-counter medications and vitamin supplements, and blood and urine samples were collected from 354 men during 2003–2004. Blood was collected into red-top tubes and allowed to clot for 20–30 min. Serum for exposure analyses was transferred to hexane-rinsed glass tubes and frozen. Serum and urine samples for hormone assays were frozen in polypropylene tubes. In addition, stored serum samples collected from 51 men in 2001–2003 were analyzed for hormone and exposure levels. The study protocol was approved by institutional review boards at the University of Wisconsin, Madison, and the University of Illinois at Chicago, and all subjects gave written informed consent before participation.

### Exposure analyses

Serum samples were tested for PBDEs, PCBs, and DDE by the Wisconsin State Laboratory of Hygiene as previously described (Anderson et al. 2008). Briefly, sera were extracted with hexane/ethyl ether, with cleanup and fractionation using Florisil, silica gel, and concentrated sulfuric acid. PBDEs were analyzed by gas chromatography–mass spectrometry (GC-MS), and PCBs and DDE by GC. Quality control was monitored by the use of method blanks, spikes of bovine serum, duplicates of bovine serum spikes or sample duplicates, surrogate spikes, and confirmation of the analytes by second column or GC-MS, as appropriate. Mean recoveries were 76–91% for 24 tri- to decaBDE congeners, 97% for DDE, and 81–94% for di- to hexaPCB congeners.

### Hormone analyses

Hormone assays were performed on serum and urine samples at Northwestern University in R.C.’s laboratory. Total T_4_ (serum and urine), total T_3_, and the free unbound concentrations of these thyroid hormones were measured by radioimmunoassay (Diagnostic Products Corporation, Inc., Los Angeles, CA). Specificity was > 99%. Interassay and intraassay coefficient of variations (CVs) were, respectively, 3.0% and 3.3% for total T_3_, 4.0% and 5.0% for total T_4_, 6.9% and 4.3% for free T_4_, 28.8% and 7.9% for free T_3_, and 5.6% and 14.9% for urinary total T_4_. We measured rT_3_ in a competitive radioimmunoassay with a sensitivity of 7.0 ng/dL (ALPCO Diagnostics, Windham, NH). The antiserum used was highly specific: T_3_ and T_4_ cross-react by < 0.1%. Interassay and intraassay CVs were 13.4% and 5.5%, respectively.

We measured TSH and TBG in the Immulite System (Diagnostic Products). The TSH assay had a sensitivity of 0.002 μIU/mL and was highly specific, with < 0.1% cross-reaction with other glycoprotein hormones. The TBG assay was also highly specific, with a sensitivity of 1.1 μg/mL. Interassay CVs were 14.7% for TSH and 9.7% for TBG.

We examined the distribution of T_4_ binding in plasma by radioelectrophoresis (Borst et al. 1982; Leopold et al. 1987). We separated albumin- and TBG-bound ^125^I-T_4_ on agarose gels after incubation of ^125^I-T_4_ with the serum for 2 hr at 37°C. TTR, which we did not quantify in this analysis, is clearly separated from TBG in this system. The gels were stained with bromothymol blue to identify albumin in the samples. Standards of TBG and TTR were run in parallel to determine the location relative to albumin on the gel. The areas corresponding to TBG and albumin were cut out of the gel and counted in a gamma counter, and the percentage of the total ^125^I-T_4_ in each fraction was determined. Interassay CVs were 3.4% for TBG-bound T_4_ and 11.9% for albumin-bound T_4_.

We measured urine creatinine spectrophotometrically by the Jaffe reaction after ethyl ether extraction. Interassay and intra-assay CVs were 9.3% and 5.6%, respectively.

We measured testosterone in serum using a coated tube assay that employs ^125^I-labeled testosterone as the tracer (Diagnostic Systems Laboratories, Webster, TX). The antiserum cross-reacted < 0.9% with androstenedione and androstenediol and 5.8% with dihydrotestosterone. Interassay and intraassay CVs were 17.0% and 6.6%, respectively. We measured SHBG using a competitive radioimmunoassay with a sensitivity of 5 nmol/L (Diagnostic Systems Laboratories). The interassay and intraassay CVs were 15.7% and 6.6%, respectively.

SHBG-bound testosterone was determined as described by Bonfrer et al. (1989). We equilibrated a 0.2-mL volume of serum diluted 1/8 with buffer with ^3^H-estradiol overnight at 4°C. A 0.10 mL suspension of a conconavalin-A (Con-A) Sepharose conjugate was added to the serum. SHBG was allowed to bind to the Con-A during a 30-min incubation period at room temperature. Testosterone in the serum maintains its equilibrium concentration with SHBG in the presence of endogenous factors such as other androgens, estrogens, and free fatty acids (Bonfrer et al. 1989; Street et al. 1989). Separation of unbound ^3^H-testosterone from that bound to the Sepharose Con-A was achieved by centrifugation at 0°C to minimize dissociation of bound estradiol. The interassay and intraassay CVs were 4.5% and 3.6%, respectively.

Thyroglobulin antibodies and hemoglobin A1c (HA1c) were measured by Quest Diagnostics (Auburn Hills, MI, and Wood Dale, IL). HA1c was measured by affinity chromatography, which measured total glycosylated hemoglobin, from which HA1c is calculated. Thyroglobulin antibodies were detected in an immunochemiluminometric assay that used avidin beads, biotinylated thyroglobulin, and acridinium ester–labeled thyroglobulin. Total cholesterol and triglycerides were measured by Quest Diagnostics for samples collected in 2004–2005 and by Meriter Laboratories (Madison, WI) for samples collected in 2001–2003. Total serum lipids were calculated by the following formula: total cholesterol (mg/dL) × 2.27 + triglycerides (mg/dL) + 62.3.

### Statistical analyses

For results below the limit of detection (LOD), we imputed BDE and PCB congener concentrations as the LOD for the individual congener divided by 2. We summed BDE congeners 28, 47, 49, 85, 99, 100, 138, and 153 to derive ∑PBDEs. Similarly, ∑PCB included PCB congeners 66, 74, 99, 118, 128, 146, 167, 172, 177, 178, 180, 183, 193, 194, 201, and 206, as well as coeluting congeners 163/138, 170/190, 203/196, 202/171, 208/195, 187/182, and 132/153/105. We used natural log transformations (ln) of ∑PBDEs, BDE-47, ∑PCBs, DDE, TSH, rT_3_, free T_3_, urinary T_4_, and SHBG to approximate a normal distribution.

We explored associations of thyroglobulin antibodies and thyroid disease with ∑PBDEs greater than the 90th or 95th percentiles in the full cohort of 405 men using logistic regression models, with adjustment for age.

Participants were excluded from the hormone analyses if they reported medical conditions or medication use known to affect thyroid hormone levels (Meier and Burger 2005). Complete data for exposure and hormone measures were available for 308 men for the hormone analysis after excluding participants missing data for lipids (*n* = 12); using thyroid hormones or having thyroid disease (*n* = 21); using blood-glucose–regulating medications or having diabetes (*n* = 60); using other hormones (*n* = 11; testosterone, systemic corticosteroids, melatonin, human growth hormone); or using other medications known to affect thyroid hormones (*n* = 4; dilantin, tegretol, lithium, carbodopa).

Associations of hormones with ∑PBDEs and BDE-47 were modeled using linear regression, and Pearson’s partial correlation coefficients for associations of hormones with exposures were estimated with the same variables used in the linear regression models. We considered age, body mass index (BMI), and serum lipids to be important covariates and included them in all multivariate models. Other potential confounding variables were added individually to these models to determine if their inclusion affected the conclusion about the significance of the PBDE/hormone association (*p* < 0.05 or *p* > 0.05). Factors that were evaluated as potential confounders included smoking, alcohol use, medication use (antilipids, beta blockers, furosamide), Great Lakes sport fish meals in the past year, sport fish meals in the past year, ∑PCBs, DDE, years consuming sport fish meals, years consuming Great Lakes sport fish meals, and HA1c level. We also considered measured levels of testosterone, SHBG, and SHBG-bound testosterone as potential confounders for thyroid hormone analyses.

We examined modification of the effect of ∑PBDEs on hormones by other exposure covariates (all potential confounding variables noted above) in linear regression models that included multiplicative interaction terms for ∑PBDEs and the potential effect modifier, adjusting for age, BMI, and lipids. We did not evaluate covariates identified as effect modifiers (*p* < 0.05 for interaction term) as potential confounders, but we stratified models of the effects of ∑PBDEs on hormones by above and below median levels of the effect modifier.

To determine if results were affected by extreme hormone values, we estimated models after exclusion of participants with values more than three interquartile ranges above the 75th percentile or below the 25th percentile for hormone measures. Models were also reestimated using a variable for ∑PBDEs where congeners below the LOD were imputed as 0, but this did not affect our findings.

We designed this study to explore associations of PBDEs with standard thyroid hormone parameters, free and total T_4_ and T_3_, as well as TSH, and also with additional laboratory parameters to test specific mechanisms of action. We explored patterns in the associations of PBDEs with thyroid hormones regarding congener-specific associations and independence of associations to examine mechanistic hypotheses.

We estimated dose–response models by linear regression for BDE congeners 47, 99, 100, and 153 using either indicator variables for tertiles 2 and 3, with tertile 1 as the reference category, or the ordinal tertile variable to test for a trend over the categories. For BDEs 99, 100, and 153, the lowest tertile included all participants with measurements < LOD. BDE tertiles (ng/g) were defined as follows: BDE-47, < LOD to 0.08 (*n* = 106), > 0.08–0.15 (*n* = 101), > 0.15 (*n* = 101); BDE-99, < LOD (*n* = 117), 0.025–0.046 (*n* = 97), > 0.046 (*n* = 94); BDE-100, < LOD (*n* = 205), 0.026–0.05 (*n* = 52), > 0.05 (*n* = 51); BDE-153, < LOD (*n* = 212), 0.05–0.099 (*n* = 49), > 0.099 (*n* = 47). We examined similar models for ∑PBDE quartiles.

Because an effect of PBDEs at one point in thyroid homeostasis could potentially change other related thyroid hormone parameters, we examined the independence of significant associations of thyroid hormones with ∑PBDEs regarding other measured thyroid hormones. When we identified a significant association between ∑PBDEs and a thyroid hormone, we further adjusted the linear regression model for other thyroid hormone levels individually. When the β-coefficient for ∑PBDEs changed by > 20% after adjustment for a second hormone, this suggested that the effect of ∑PBDEs on the original hormone may be related to or mediated by the second hormone.

## Results

Characteristics of the cohort included in the hormone analysis are shown in Table 1. Most men drank alcohol at least once a month (78%), but few smoked cigarettes (11%), and medication use varied, with 33% using anti-lipidemics, 17% using beta blockers, and 3% using furosamide (data not shown). Levels of ∑PBDEs in the men were similar to those found for a large sample representative of the U.S. population of similar age and ethnicity: in this study, geometric mean = 27 ng/g lipid [95% confidence interval (CI), 24–30 ng/g lipid]; in the National Health and Nutrition Examination Survey, geometric mean = 34 ng/g lipid (95% CI, 27–43 ng/g lipid) (Anderson et al. 2008). However, levels of ∑PCBs were somewhat higher in the present study than in the National Health and Nutrition Survey (Anderson et al. 2008). Because we excluded men with thyroid disease, thyroid hormone levels were predominantly within normal ranges (Table 1).

∑PBDEs was significantly and positively associated with several thyroid hormones, including total T_4_, free T_4_, urinary T_4_, rT_3_, and albumin-bound T_4_ (only after exclusion of two extreme outliers) and was negatively associated with TSH, but only after control for sport fish consumption (Table 2). We found generally similar associations for these thyroid hormones with BDE-47, the dominant BDE congener (Table 2).

**Figure 1:**
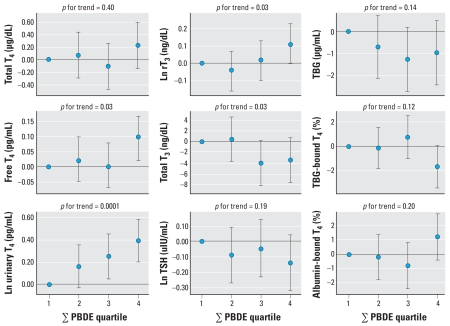
β-Coefficients and 95% CIs from regression models for associations of individual ∑PBDE quartiles with hormone levels. *p*-Values are from regression models for associations of ordinal ∑PBDE quartile variables with hormone levels. All models adjusted for age, BMI, and serum lipids. Urinary total T_4_ was also adjusted for urinary creatinine.

Figure 1 shows dose–response models for quartiles of ∑PBDEs. We saw the strongest dose response for urinary T_4_, whereas only the highest ∑PBDE quartile was elevated for free T_4_ and rT_3_. Total T_3_, which was not significantly associated in the continuous analysis (Table 2), was significantly negatively associated with ∑PBDE quartiles. On the other hand, total T_4_ and TSH were not significantly associated with ∑PBDEs in the ordinal dose–response models. The effect of ∑ PBDEs on T_4_ binding to serum proteins was limited to the highest exposure quartile.

Urinary T_4_ was the only hormone associated with all four BDE congeners (Table 3). rT_3_, total T_4_, and free T_4_ were positively associated with BDE-99 and BDE-153, total T_3_ was negatively associated with BDE-47 and BDE-153, and free T_3_ was negatively associated with BDE-153. BDE-100 was negatively associated with TBG-bound T_4_ and positively associated with albumin-bound T_4_, with similar associations for BDE-153, but only in the highest tertile (Table 3).

We found significant associations among many of the thyroid hormone measurements (data not shown). Because an effect of PBDEs at one point in thyroid homeostasis could potentially change other related thyroid hormone parameters, we examined the independence of significant associations of thyroid hormones with ∑PBDEs regarding other measured thyroid hormones (Table 4). The associations of urinary T_4_ and albumin-bound T_4_ with ∑PBDEs were independent of other thyroid hormones (Table 4). However, associations of ∑PBDEs with rT_3_, free T_4_, total T_4_, total T_3_, and TSH were independent of many but not all other thyroid hormone levels.

The effect of PBDEs on rT_3_, free T_4_, TBG-bound T_4_, and albumin-bound T_4_ was significantly modified by HA1c levels, with stronger associations in persons with higher HA1c levels (data not shown). Effects of PBDEs on rT_3_ and albumin-bound T_4_ were stronger among infrequent sport fish consumers, whereas effects on TSH were stronger among frequent consumers (data not shown). We found no evidence of effect modification by PCBs or DDE, years of sport fish consumption, medication use, age, BMI, serum lipids, smoking, alcohol use, or steroid hormone levels.

Testosterone, SHBG, and SHBG-bound testosterone were not associated with ∑PBDEs or BDE-47 as a continuous variable (Table 2), but the ordinal variable for BDE-47 tertiles was positively associated with testosterone (Table 3).

We studied thyroid disease and thyroglobulin antibodies in the entire cohort of 405 men. Thyroglobulin antibodies were present in 7.8% of the full cohort and in 31.3% of those whose ∑PBDEs exceeded the 95th percentile [odds ratio (OR) = 6.1; Table 5]. High PBDE exposure was not significantly associated with a diagnosis of thyroid disease (Table 5).

## Discussion

Exposure to PBDEs at levels comparable with those in the general U.S. population was associated with thyroid and steroid hormone levels in adult men without thyroid disease or diabetes. PBDEs were positively related to measures of T_4_ (total T_4_, free T_4_, urinary T_4_) and rT_3_, and inversely related to total T_3_ and TSH. PBDEs were positively related to the percentage of T_4_ bound to albumin and inversely related to the percentage of T_4_ bound to TBG. Associations of BDE congeners with thyroid hormones varied. BDE-47 was positively associated with testosterone levels.

Our finding of increased thyroglobulin antibodies in 31% of participants with the highest PBDE body burdens is potentially of biologic significance because thyroglobulin antibodies are found in 80–90% of patients with chronic autoimmune thyroiditis and 50–60% of patients with Grave’s disease (Marcocci and Marino 2005). The 8% prevalence of antibodies in the entire cohort is similar to rates seen in normal adult male populations (Hollowell et al. 2002). Exposure to PCBs, which are similar in structure to PBDEs, has been associated with increased antithyroperoxidase antibodies (Langer et al. 2007). The small number of cases of hypo- and hyperthyroid disease limit our ability to draw conclusions on effects of PBDEs on thyroid disease, but the thyroglobulin results may indicate an increased susceptibility to autoimmune thyroiditis in PBDE-exposed persons.

To our knowledge, epidemiologic data on the effects of PBDEs on thyroid hormones in adults is limited to four published studies. First, a longitudinal study of 11 electronic recycling employees found no significant associations of BDE congeners with TSH, total T_3_, or free T_4_, but did note nonsignificant trends for increasing free T_4_ with BDEs 28, 153, and 183 (Julander et al. 2005). Second, free T_4_ and TSH were not significantly associated with PBDEs in 36 New York anglers, although the associations of BDE congeners with free T_4_ were consistently positive (Bloom et al. 2008), and the authors estimated that 318 persons would be required to reach significance for the association of ∑PBDEs with free T_4_. Third, Hagmar et al. (2001) found a significant negative association of BDE-47 with TSH but no significant association with free and total T_3_ and T_4_ in 110 men with high consumption of fish from the Baltic Sea. Our results are consistent with the decreased TSH in Hagmar et al.’s study and with the positive direction of the free T_4_ associations of Bloom et al. (2008) and Julander et al. (2005). Fourth, Yuan et al. (2008) found higher TSH levels in electronic waste workers compared with unexposed persons, but PBDE exposures levels were substantially higher in that study than in our fish consumer cohort.

Our findings of a positive association of PBDEs with T_4_ and free T_4_ are not, however, consistent with results of laboratory animal studies. In rats and mice, PBDE mixtures and BDE-47 have been shown to decrease T_4_ and free T_4_ (Hallgren et al. 2001; Hallgren and Darnerud 2002; Stoker et al. 2004; van der Ven et al. 2008; Zhou et al. 2001, 2002). T_3_ was also decreased in some studies, but to a lesser extent than total T_4_ (Zhou et al. 2001), and TSH was not affected, except in a 31-day exposure in male rats that had decreased TSH (Stoker et al. 2004). It is not clear why our results are inconsistent with decreased T_4_ found in PBDE-exposed laboratory animals. Thyroid hormone regulation is similar in vertebrates, but some functions differ by species. For example, more T_3_ is produced by the thyroid in rats than in that of humans (40% vs. 20%), increasing the importance of deiodinases in controlling T_3_ levels in humans. In addition, TTR is the dominant binding protein in rats, whereas most thyroid hormone circulates bound to TBG in humans. Rats are more sensitive to effects of PBDEs on thyroid hormones than are mice (Hallgren et al. 2001). Inconsistencies could also be related to generally higher exposure levels in animals, younger life stage at exposure, and congener-specific effects. Mice exposed to BDE-209 had decreased T_3_ but not T_4_ (Tseng et al. 2008). Finally, there may be substantial differences in the effects of acute versus chronic exposure.

A major strength of our study is the measurement of specific hormones and BDE congeners, which may offer insights into potential biological pathways. The analysis of the independence of associations between thyroid hormones and PBDEs regarding other measured thyroid hormones suggests independent path-ways for PBDE effects on urinary T_4_ levels and T_4_ serum protein binding proportions, whereas changes in rT_3_, total T_4_, free T_4_, total T_3_, and TSH were interrelated. BDE-congener–specific relationships also support different pathways, with associations of BDEs 47, 99, 100, and 153 with urinary T_4_, BDEs 100 and 153 with T_4_ serum protein binding proportions, and BDEs 99 and 153 with rT_3_, total T_4_, and free T_4_.

The association of PBDEs with rT_3_ suggests that PBDEs may affect thyroid hormone deiodinases. Deiodinases play a key role in control of cellular levels of T_3_ (Bianco and Kim 2006). D_2_ deiodinase removes iodide from outer ring of thyroid hormones (*meta* position), converting T_4_ to T_3_, whereas D_3_ deiodinase removes an iodide from the inner ring (*ortho* position) converting T_4_ to rT_3_. D_1_ can remove iodide from the inner and outer rings of thyroid hormones. Changes in deiodinase activity can affect circulating hormone levels, as demonstrated by studies of mice carrying deletion mutations. For example, mice carrying deletion mutations in D_2_ have elevated T_4_ and TSH but no changes in T_3_ (Schneider et al. 2001), those with D_1_ mutations have elevated T_4_ and rT_3_ but no change in T_3_ and TSH (Schneider et al. 2006), whereas those with D_3_ mutations are hypothyroid with decreased T_4_ and T_3_ but no change in TSH (Hernandez et al. 2006). These studies suggest that inhibition of outer ring deiodinases, most likely D_1_, by PBDEs could account for the increased T_4_ and rT_3_ in our participants with higher exposures. A possible mechanism is competitive inhibition of outer ring deiodinase by BDEs. Evidence that outer ring deiodinases in fish may debrominate BDE-99 to BDE-47 by removal of a bromine from the *meta* position (Benedict et al. 2007), as well as our finding that BDEs 99 and 153, both of which have a bromine in the *meta* position, were positively associated with rT_3_, free T_4_, and total T_4_, supports this hypothesis. The negative relationship of PBDEs with TSH might be a normal feedback response to elevated T_4_ levels. Decreased production of total T_3_ could also be a consequence of decreased outer ring deiodinase activity, although mice with outer ring deiodinase deletion mutations did not have abnormal T_3_ levels.

The strongest PBDE association we observed was related to urinary total T_4_ levels. Urinary total T_4_ levels are not routinely assessed clinically. The increase we found, however, is consistent with the noted increases in serum free T_4_ and albumin-bound T_4_, although the association of urinary T_4_ with PBDEs was independent of serum free T_4_ and albumin-bound T_4_. Future studies could assess effects of PBDEs on urinary thyroid hormone metabolites as a potential mechanism.

The associations of PBDEs with T_4_ serum protein binding proportions suggest that PBDEs could be displacing T_4_ from TBG. Hydroxylated BDE metabolites were able to bind to TTR *in vitro* (Hamers et al. 2006), and TTR in serum from BDE-47–treated rats showed decreased binding to ^125^I-T_4_ serum compared with serum from untreated rats (Hallgren and Darnerud 2002). However, to our knowledge, the potential for BDE congeners and metabolites to compete with T_4_ binding to TBG has not been tested.

Steroid hormones can affect thyroid hormones through changes in TBG production. We did not find that testosterone levels modified the effects of PBDEs on thyroid hormones, but testosterone and SHBG did confound several associations of PBDEs and thyroid hormones. In addition, we found a positive association of testosterone with BDE-47. Hagmar et al. (2001) did not find associations of BDE-47 with free testosterone, follicle-stimulating hormone, luteinizing hormone, or prolactin in men. In male rats, the onset of prenuptial separation was delayed and ventral prostate and seminal vesicle weights were decreased, but luteinizing hormone and testosterone were not changed by PBDE exposure (Stoker et al. 2004). However, Stoker et al. (2005) found increased luteinizing hormone and a trend for increased steroid concentrations in PBDE-exposed adult male rats, and Lilienthal et al. (2006) observed that testosterone was decreased in male pups pre-natally exposed to BDE-99. BDE congeners, in particular BDE-100, are androgen antagonists *in vitro* (Hamers et al. 2006; Stoker et al. 2005).

Although we excluded persons with diabetes from the hormone analyses, our data suggest that the effects of PBDEs on rT_3_, free T_4_, and albumin- and TBG-bound T_4_ are stronger in persons with higher HA1c levels, which could place persons with moderately increased blood glucose at higher risk of thyroid hormone disruption by PBDEs. rT_3_ is increased by fasting, malnutrition, and poorly controlled diabetes. Alternatively, changes in thyroid hormones may affect blood glucose (Chidakel et al. 2005).

Our results also suggest that fish consumption may modify the effect of PBDEs on thyroid function. We saw a stronger effect of PBDEs on rT_3_ and albumin-bound T_4_ among infrequent consumers and stronger effects on TSH among frequent sport fish consumers. Furthermore, some associations of PBDEs with hormones were modified by consumption of sports fish, but not by PCB or DDE body burdens. These findings are consistent with an interaction between PBDEs and other contaminants in fish on thyroid hormones.

The strengths of the present study include the use of a large, well-defined cohort; assessment of multiple hormones; and consideration of other environmental exposures that can affect thyroid hormones. Our conclusions are limited by those of any cross-sectional investigation. Although our results are inconsistent with animal studies, they are consistent with several human studies. The associations we found were relatively weak, and the highest proportion of hormone variation explained by PBDE was approximately 6% for urinary T_4_ (*r* = 0.25). There were some inconsistencies between results of models with continuous and ordinal exposure variables, with effects seen only in highest exposure category for some hormones. This pattern might be related to the extremely skewed distribution of PBDEs in the study cohort (Table 1). In addition, some hormone parameters show inconsistencies between models of ∑PBDEs and individual BDE congeners, which may be explained by congener-specific effects, as supported by animal and *in vitro* data.

Although PBDE levels are lower than PCB or DDE levels, PBDE body burdens are increasing (Schecter et al. 2005). Older adults, who have a high risk of thyroid disease, are more likely to have BDE-47 levels above the 95th percentile level of 291 ng/g lipid (Sjodin et al. 2008). In the present study, exposures were similar to those of the U.S. population (Anderson et al. 2008). With increasing PBDE body burdens, we found increases in T_4_, but decreases in T_3_ and TSH. In addition, thyroglobulin antibodies were higher in men with the highest PBDE body burdens. This is the first large study to link PBDE exposure with changes in thyroid antibodies and thyroid hormone homeostasis in men.

## Figures and Tables

**Table 1:** Distribution of covariates, exposure measures, and endogenous hormone levels in 308 men.

			Percentile				
Characteristic	Mean	Minimum	25th	50th	75th	95th	Maximum
Age (years)	59	30	53	59	67	74	82
BMI (kg/m^2^)	29.8	18.4	26.8	29.2	32.0	38.4	50.2
Serum lipids (mg/dL)	720.8	370.1	600.1	693.6	810.0	976.6	2459.1
Urinary creatinine (μg/mL)	1402.4	65.8	843.3	1268.1	1877.3	2753.8	7290.3
HA1c (%)	5.6	4.4	5.4	5.6	5.9	6.5	8.8
∑PBDEs^a^ (ng/g lipid)	69.9	15.8	29.3	38.4	62.4	193.4	1360.2
∑PBDEs^a^ (ng/g)	0.47	0.13	0.20	0.26	0.41	1.49	10.15
BDE-47^a^ (ng/g)	0.22	0.01	0.07	0.11	0.18	0.89	5.90
BDE-99^a^ (ng/g)	0.06	0.01	0.01	0.03	0.05	0.16	2.60
BDE-100^a^ (ng/g)	0.04	0.01	0.01	0.01	0.03	0.17	0.87
BDE-153^a^ (ng/g)	0.08	0.03	0.03	0.03	0.07	0.30	2.30
∑PCBs^a^ (ng/g)	4.10	1.17	2.04	2.99	5.03	10.72	28.12
DDE^a^ (ng/g)	3.29	0.08	1.20	2.10	4.00	10.00	20.00
Years eating sport fish	38	0	25	40	50	62	70
Years eating Great Lakes sport fish	32	0	20	33	50	60	70
Sport fish meals in last 12 months	29	0	4	18	40	104	265
Great Lakes sport fish meals in last 12 months	23	0	2	14	34	72	156
TSH^b^ (μIU/mL)	1.82	0.29	1.04	1.55	2.25	4.00	9.30
Total T_3_^b^ (ng/dL)	99.0	55.6	89.5	99.0	108.1	123.3	145.9
Free T_3_^b^ (pg/mL)	2.28	0.85	1.67	2.08	2.64	3.91	11.02
rT_3_ (ng/dL)	25.9	9.1	18.3	22.7	27.0	41.0	245.5
Total T_4_^b^ (μg/dL)	7.2	3.3	6.4	7.1	8.0	9.0	10.6
Free T_4_^b^ (ng/mL)	1.19	0.62	1.01	1.19	1.35	1.60	1.82
Urinary Total T_4_ (pg/mL)	1216.9	68.6	581.5	1084.7	1522.5	3033.3	5567.7
TBG^b^ (μg/mL)	19.1	1.5	16.4	18.8	21.0	27.0	43.7
TBG-bound T_4_ (%)	77.3	49.5	74.4	77.7	80.8	84.4	90.9
Albumin-bound T_4_ (%)	17.7	5.9	14.7	17.2	20.2	25.9	46.2
Testosterone (ng/mL)	3.08	0.10	2.28	2.93	3.85	5.24	6.33
SHBG (nmol/L)	169.6	0	88.3	142.4	221.2	421.5	630.0
SHBG-bound testosterone (%)	32.5	0.2	27.5	32.0	37.5	44.7	54.4

a For BDE and PCB congeners and DDE, we imputed values < LOD as the LOD for each analyte/2: LOD = 0.025 ng/g for BDEs 28, 47, 49, 85, 99, and 100; LOD = 0.05 ng/g for BDEs 138 and 153. Proportion of samples > LOD: BDE-47 = 98%, BDE-99 = 62%, BDE-100 = 33% and BDE-153 = 31%.

b Normal reference range: TSH = 0.5–4.7 μIU/mL, total T_3_ = 70–195 ng/dL, free T_3_ = 1–4.2 pg/mL, total T_4_ = 5–12 μg/dL, free T_4_ = 0.8–2 ng/mL, TBG = 13–39 μg/mL.

**Table 2:** Associations of hormones with ∑PBDEs and BDE-47: Pearson’s correlation coefficients.

			∑PBDEs		BDE-47	
Hormone	No.	Measure	Unadjusted	Adjusted^a^	Unadjusted	Adjusted^a^
Ln TSH (μIU/mL)	304	*r*-Value	−0.05	−0.10^b^	−0.08	−0.14
		*p*-Value	0.39	0.07	0.18	0.02
Total T_3_ (ng/dL)	305	*r*-Value	−0.02	−0.04	−0.02	−0.04
		*p*-Value	0.68	0.44	0.79	0.51
Ln free T_3_ (pg/mL)	306	*r*-Value	−0.05	−0.06	−0.003	−0.01
		*p*-Value	0.35	0.32	0.95	0.90
Ln rT_3_ (ng/dL)	304	*r*-Value	0.22	0.14	0.21	0.12^c^
		*p*-Value	< 0.0001	0.02	0.0003	0.04
Total T_4_ (μg/dL)	307	*r*-Value	0.10	0.12^d^	0.07	0.09
		*p*-Value	0.07	0.03	0.21	0.12
Free T_4_ (ng/mL)	308	*r*-Value	0.13	0.16	0.09	0.13
		*p*-Value	0.03	0.005	0.12	0.03
Ln urinary total T_4_ (pg/mL)^e^	268	*r*-Value	0.20	0.25	0.19	0.25
		*p*-Value	0.001	< 0.0001	0.002	< 0.0001
TBG (μg/mL)	303	*r*-Value	0.04	0.05	0.02	0.02
		*p*-Value	0.46	0.39	0.78	0.73
TBG-bound T_4_ (%)	267	*r*-Value	−0.08	−0.11	−0.07	−0.11^f^
		*p*-Value	0.18	0.06	0.27	0.08
Albumin-bound T_4_ (%)	267	*r*-Value	0.07	0.11^g^	0.05	0.11^g^
		*p*-Value	0.26	0.06	0.37	0.08
Testosterone (ng/mL)	307	*r*-Value	−0.06	−0.01	−0.01	0.06
		*p*-Value	0.26	0.86	0.81	0.28
Ln SHBG (nmol/L)	269	*r*-Value	0.04	0.04	0.05	0.05
		*p*-Value	0.47	0.54	0.41	0.44
SHBG-bound testosterone (%)	269	*r*-Value	−0.02	−0.04	−0.03	−0.05
		*p*-Value	0.73	0.49	0.62	0.38

a Adjusted for age, BMI, and serum lipids. Unless otherwise noted, significance of adjusted models did not change with further adjustment for the following covariates (added individually to model): smoking, alcohol use, antilipid medications, beta blocker medications, furosamide medication, Great Lakes sport fish meals in the past year, sport fish meals in the past year, ln ∑PCBs, ln DDE, years consuming sport fish meals, years consuming Great Lakes sport fish meals, and HA1c level. Unless otherwise noted, significance of adjusted models for thyroid hormones did not change with further adjustment for testosterone level, ln SHBG level, and SHBG-bound testosterone level.

b Significant with further adjustment for Great Lakes fish meals or sport fish meals in the last year (*r* = −0.12, *p* = 0.04).

c Borderline significant (0.05 < *p* < 0.10) with further adjustment for HA1c, ln SHBG, SHBG-bound testosterone, or ln DDE

d Borderline significant (0.05 < *p* < 0.10) with further adjustment for alcohol consumption, Great Lakes fish meals in the last year, ln SHBG, or SHBG-bound testosterone.

e Adjusted for urinary creatinine.

f Significant with further adjustment for testosterone (*r* = −0.12, *p* = 0.05).

g Significant after exclusion of two extreme outliers for T_4_-bound albumin (*r* = 0.13, *p* = 0.03).

**Table 3:** Associations of hormones with BDE-47, BDE-99, BDE-100, and BDE-153 tertiles.

Hormone	Measure	BDE-47	BDE-99	BDE-100	BDE-153
Ln TSH (μIU/mL)	β for tertile 2^a^	−0.14*	−0.07	−0.05	0.06
	β for tertile 3	−0.14*	−0.10	−0.06	−0.02
	*p*-Value for trend^b^	0.08	0.27	0.42	0.96
Total T_3_ (ng/dL)	β for tertile 2	−2.49	−2.78	−5.61**	−3.34
	β for tertile 3	−4.33**	−0.21	−1.27	−3.83*
	*p*-Value for trend	0.02	0.84	0.18	0.04^c^
Ln free T_3_ (pg/mL)	β for tertile 2	−0.04	0.01	−0.004	0.03
	β for tertile 3	−0.07	−0.03	−0.05	−0.15**
	*p*-Value for trend	0.18	0.55	0.47	0.04^d^
Ln rT_3_ (ng/dL)	β for tertile 2	0.03	0.01	−0.04	0.04
	β for tertile 3	0.09*	0.13**	0.11**	0.12**
	*p*-Value for trend	0.06^e^	0.009	0.11	0.03^f^
Total T_4_ (μg/dL)	β for tertile 2	−0.17	−0.15	−0.25	0.13
	β for tertile 3	−0.01	0.32**	0.24	0.38**
	*p*-Value for trend	0.92	0.06^g^	0.44	0.04^h^
Free T_4_ (ng/mL)	β for tertile 2	−0.004	0.02	−0.03	0.03
	β for tertile 3	0.04	0.10**	0.07**	0.10**
	*p*-Value for trend	0.25	0.002	0.13	0.009
Urinary total T_4_ (pg/mL)^i^	β for tertile 2	0.02	−0.03	0.06	0.03
	β for tertile 3	0.30**	0.30**	0.24**	0.20**
	*p*-Value for trend	0.0007	0.0009	0.01	0.06^j^
TBG (μg/mL)	β for tertile 2	−0.32	−1.80**	−1.17*	−0.56
	β for tertile 3	−0.69	−0.68	0.19	0.07
	*p*-Value for trend	0.28	0.22	0.79	0.88
TBG-bound T_4_ (%)	β for tertile 2	0.03	−1.05	−2.14**	1.23
	β for tertile 3	−1.29*	−0.77	−1.76**	−1.78**
	*p*-Value for trend	0.10^k^	0.29	0.008	0.17
Albumin-bound T_4_ (%)	β for tertile 2	−0.59	0.93	1.67**	−0.94
	β for tertile 3	1.03	0.65	1.52**	1.58**
	*p*-Value for trend	0.16	0.34	0.02	0.15
Testosterone (ng/mL)	β for tertile 2	0.32**	0.33**	−0.11	0.31*
	β for tertile 3	0.36**	0.21	−0.18	−0.25
	*p*-Value for trend	0.02^l^	0.16	0.25	0.46
Ln SHBG (nmol/L)	β for tertile 2	−0.13	−0.20*	−0.08	0.10
	β for tertile 3	0.05	−0.01	0.03	−0.09
	*p*-Value for trend	0.64	0.88	0.99	0.66
SHBG-bound testosterone (%)	β for tertile 2	−1.49	−1.81*	1.49	1.02
	β for tertile 3	−1.24	−1.38	−0.34	−0.64
	*p*-Value for trend	0.22	0.16	0.90	0.81

a β-Coefficient estimate from linear regression for association of BDE tertile with hormone level, adjusted for age, BMI, and serum lipids. Unless otherwise noted, significance of adjusted models did not change with further adjustment for the following covariates (added individually to model): smoking, alcohol use, antilipid medications, beta blocker medications, furosamide medication, Great Lakes sport fish meals in the past year, sport fish meals in the past year, ln ∑PCBs, ln DDE, years consuming sport fish meals, years consuming Great Lakes sport fish meals, and HA1c level. Unless otherwise noted, significance of adjusted models for thyroid hormones did not change with further adjustment for testosterone level, ln SHBG level, and SHBG-bound testosterone level.

b *p*-Value from linear regression model for ordinal BDE tertile variable indicating trend over BDE tertiles.

c Borderline significant (0.05 < *p* < 0.10) with further adjustment for years consuming sport fish or years consuming Great Lakes sport fish.

d Borderline significant (0.05 < *p* < 0.10) with further adjustment for smoking, alcohol use, or years consuming Great Lakes sport fish.

e Significant with exclusion of extreme hormone outliers or with further adjustment for smoking.

f Borderline significant (0.05 < *p* < 0.10) with further adjustment for HA1c, ln SHBG, or SHBG-bound testosterone.

g Significant with further adjustment for years consuming sport fish or Great Lakes sport fish.

h Borderline significant (0.05 < *p* < 0.10) with further adjustment for HA1c, ln SHBG, SHBG-bound testosterone, smoking, alcohol use, or Great Lake sport fish meals in last year.

i Adjusted for urinary creatinine.

j Significant with further adjustment for years consuming sport fish or beta blocker use.

k Significant with further adjustment for testosterone levels.

l Borderline significant (0.05 < *p* < 0.10) with further adjustment for HA1c or alcohol use.

* Individual tertile 0.05 < *p* < 0.10.

** Individual tertile *p* < 0.05.

**Table 4:** Associations of thyroid hormones with ∑PBDEs: confounding of significant associations by other thyroid hormones.

		Association of thyroid hormone with ∑PBDEs with adjustment^a^						
Hormone	Unadjusted	Urinary T_4_	Albumin-bound T_4_	TSH	Total T_4_	Free T_4_	rT_3_	Total T_3_
Ln urinary T_4_								
β b	0.21	—	0.21	0.20	0.20	0.20	0.21	0.22
*p*-Value^b^	0.001	—	0.0001	0.001	0.001	0.001	0.001	0.001
Albumin-bound T_4_								
β	0.79	0.94	—	0.76	0.91	0.83	0.93	0.72
*p*-Value	0.04	0.02	—	0.04	0.01	0.03	0.01	0.05
Ln TSH								
β	−0.10	−0.07*	−0.09	—	−0.08*	−0.09	−0.10	−0.10
*p*-Value	0.04	0.18	0.08	—	0.10	0.07	0.04	0.03
Total T_4_								
β	0.21	0.17	0.24	0.10*	—	0.01*	0.19	0.24
*p*-Value	0.03	0.11	0.02	0.24	—	0.88	0.05	0.008
Free T_4_								
β	0.054	0.046	0.053	0.063	0.026*	—	0.038*	0.069*
*p*-Value	0.005	0.02	0.006	0.002	0.06	—	0.04	0.003
Ln rT_3_								
β	0.071	0.067	0.076	0.067	0.043*	0.040*	—	0.067
*p*-Value	0.02	0.05	0.02	0.03	0.15	0.10	—	0.03
Total T_3_								
β	−1.50	−2.53*	−1.81*	−1.53	−1.65	−1.93*	−1.51	—
*p*-Value	0.03	0.001	0.02	0.03	0.01	0.005	0.03	—

a All linear regression were adjusted for age, BMI, and serum lipid; urinary T_4_ was also adjusted for creatinine; and TSH models were also adjusted for Great Lakes fish meals. Extreme outliers for albumin-bound T_4_ were excluded for albumin-bound T_4_ models. Ordinal variables for ∑PBDE quartiles were used in total T_3_ models.

b β-Coefficient and *p*-value for ∑PBDEs from linear regression model predicting hormone levels.

* β-Coefficient change of > 20% with control for second hormone.

**Table 5:** Age-adjusted odds of thyroid disease and thyroglobulin antibodies with high PBDE exposure in full cohort of 405 men.

		∑PBDE > 95th percentile^a^		∑PBDE > 90th percentile^b^	
Condition	All No./total (%)	No./total (%)	OR (95% CI)	No./total (%)	OR (95% CI)
Any thyroid disease^c^	20/405 (5)	1/20 (5)	1.0 (0.1−7.9)	4/40 (10)	2.4 (0.8−7.9)
Hypothyroid disease	14/405 (3.5)	0/20 (0)	—	2/40 (5)	1.7 (0.4−8.2)
Hyperthyroid disease	5/405 (1.2)	1/20 (5)	4.5 (0.5–42.9)	2/40 (5)	5.7 (0.9–36.4)
Thyroglobulin antibodies	27/348 (7.8)	5/16 (31.3)	6.1 (1.9–19.2)	5/36 (13.9)	1.9 (0.7–5.5)

a PBDE 95th percentile = 1.47 ng/g.

b PBDE 90th percentile = 0.78 ng/g.

c Any thyroid disease includes hypothyroidism, hyperthyroidism, goiter, Graves’ disease, Hashimoto’s disease, and thyroid tumor.
